# Metabolically distinct weight loss by 10,12 CLA and caloric restriction highlight the importance of subcutaneous white adipose tissue for glucose homeostasis in mice

**DOI:** 10.1371/journal.pone.0172912

**Published:** 2017-02-28

**Authors:** Laura J. den Hartigh, Shari Wang, Leela Goodspeed, Tomasz Wietecha, Barbara Houston, Mohamed Omer, Kayoko Ogimoto, Savitha Subramanian, G. A. Nagana Gowda, Kevin D. O’Brien, Karl J. Kaiyala, Gregory J. Morton, Alan Chait

**Affiliations:** 1 Department of Medicine, Metabolism, University of Washington, Seattle, Washington, United States of America; 2 Department of Medicine, Cardiology, University of Washington, Seattle, Washington, United States of America; 3 Northwest Metabolomics Research Center, Department of Anesthesiology and Pain Medicine, University of Washington, Seattle, Washington, United States of America; 4 Department of Oral Health Sciences, University of Washington, Seattle, Washington, United States of America; Universidade do Estado do Rio de Janeiro, BRAZIL

## Abstract

**Background:**

Widely used as a weight loss supplement, trans-10,cis-12 conjugated linoleic acid (10,12 CLA) promotes fat loss in obese mice and humans, but has also been associated with insulin resistance.

**Objective:**

We therefore sought to directly compare weight loss by 10,12 CLA versus caloric restriction (CR, 15–25%), an acceptable healthy method of weight loss, to determine how 10,12 CLA-mediated weight loss fails to improve glucose metabolism.

**Methods:**

Obese mice with characteristics of human metabolic syndrome were either supplemented with 10,12 CLA or subjected to CR to promote weight loss. Metabolic endpoints such as energy expenditure, glucose and insulin tolerance testing, and trunk fat distribution were measured.

**Results:**

By design, 10,12 CLA and CR caused equivalent weight loss, with greater fat loss by 10,12 CLA accompanied by increased energy expenditure, reduced respiratory quotient, increased fat oxidation, accumulation of alternatively activated macrophages, and browning of subcutaneous white adipose tissue (WAT). Moreover, 10,12 CLA-supplemented mice better defended their body temperature against a cold challenge. However, 10,12 CLA concurrently induced the detrimental loss of subcutaneous WAT without reducing visceral WAT, promoted reduced plasma and WAT adipokine levels, worsened hepatic steatosis, and failed to improve glucose metabolism. Obese mice undergoing CR were protected from subcutaneous-specific fat loss, had improved hepatic steatosis, and subsequently showed the expected improvements in WAT adipokines, glucose metabolism and WAT inflammation.

**Conclusions:**

These results suggest that 10,12 CLA mediates the preferential loss of subcutaneous fat that likely contributes to hepatic steatosis and maintained insulin resistance, despite significant weight loss and WAT browning in mice. Collectively, we have shown that weight loss due to 10,12 CLA supplementation or CR results in dramatically different metabolic phenotypes, with the latter promoting a healthier form of weight loss.

## Introduction

Obesity is reaching epidemic proportions, with more than two thirds of adults in the US characterized as overweight or obese [1]. Lifestyle modifications including exercise and caloric restriction (CR) regimens have proven efficacy for healthy weight loss, and yet obesity remains on the rise. Alternative approaches to achieving long-lasting weight loss are therefore highly sought after. Approximately 15% of US adults report the use of dietary supplements to promote weight loss [2], contributing to the billion dollar nutraceutical industry that is not heavily regulated by the FDA. As such, consumers of dietary supplements are at risk for side effects, variable efficacy, and adverse interactions with medications that may not be rigorously tested for or acknowledged by nutraceutical manufacturers. One such dietary supplement, conjugated linoleic acid (CLA), has been widely promoted for weight loss [3]. CLAs are a family of naturally occurring isomeric fatty acids, of which cis-9, trans-11 (9,11) and trans-10, cis-12 CLA (10,12) are the most naturally abundant. CLA supplements are currently formulated to contain an equal ratio of 9,11 and 10,12 CLA, but 10,12 CLA is responsible for supplemental CLA-induced weight loss [4]. While CLA has minor effects on weight loss in healthy humans [5], it has greater efficacy in individuals with obesity [6] and/or metabolic syndrome (MetS) [7]. Studies in both rodents and humans have raised safety concerns regarding CLA, which has been linked with increased inflammation and insulin resistance [8–10]. Therefore, it is important to better understand the efficacy, safety, and mechanisms by which this fatty acid mediates its effects, in contrast to weight loss achieved by CR.

Previous evidence suggests that weight loss induced by 10,12 CLA in mice [11] and humans [12] is accompanied by increased adipose tissue fatty acid oxidation (FAO), a lipid catabolic pathway uncommonly used by adipocytes. In striking contrast to the lipogenic effects of other fatty acids such as palmitate and 9,11 CLA, we and others have shown that cultured adipocytes treated with 10,12 CLA display a pro-inflammatory phenotype with increased mitochondrial respiration and FAO [13, 14]. In addition, CLA has been linked with reduced insulin signaling in cultured adipocytes [13] and mice [15], suggestive of insulin resistance. However, the mechanism whereby CLA promotes insulin resistance [16] and systemic inflammation [17], despite inducing weight loss and fat loss, has not been determined.

The current studies were undertaken to elucidate the mechanism(s) whereby 10,12 CLA supplementation promotes weight loss, with the specific goal of directly comparing this with a recommended method of achieving healthy weight loss: CR. This comparison between weight loss by 10,12 CLA versus CR could reveal novel insights regarding 10,12 CLA effects, and is an important comparison to rule out phenotypes exclusively associated with weight loss. We chose a mouse model that resembles human MetS, enabling a translational approach to targeting overweight human populations. *Ldlr*^*-/-*^ male mice fed a high fat, high sucrose + 0.15% cholesterol diet (HFHS) develop dyslipidemia, insulin resistance, and obesity [18], all components of human MetS. Mice that were then supplemented with 10,12 CLA, at a dose comparable with human CLA supplementation, exhibited weight loss associated with the preferential reduction of subcutaneous white adipose tissue (WAT). Moreover, this effect was accompanied by increased energy expenditure with enhanced subcutaneous depot-specific FAO, inflammation, and WAT browning. Despite significant weight loss, 10,12 CLA failed to improve glucose metabolism. In stark contrast, weight matched CR mice exhibited reduced WAT inflammation, maintenance of subcutaneous fat stores, and improvements in glucose metabolism. Our results suggest that relative to weight-matched CR mice, weight loss by 10,12 CLA is not metabolically healthy, supporting the need for more thorough mechanistic studies regarding the efficacy and safety of CLA.

## Methods

### Mouse study design

*Ldlr*^*-/-*^ mice on a C57Bl/6 background (Jackson Labs) were used due to their propensity to develop a phenotype resembling human metabolic syndrome, including insulin resistance, systemic inflammation, and obesity [18]. Male mice were used because they more reliably become obese and insulin resistant than female mice [19]. The study design is shown in S1 Fig. Briefly, ten-week-old male *Ldlr*^*-/-*^ mice were fed a high fat high sucrose diet (HFHS: 36% fat from lard, 36.2% sucrose, + 0.15% added cholesterol, described previously [18]) for 12 weeks (baseline), then switched to the HFHS diet with or without 9,11 or 10,12 CLA for 8 weeks to create six groups: (1) HFHS, (2) HFHS + 0.5% 9,11 CLA, (3) HFHS + 1% 9,11 CLA, (4) HFHS + 0.5% 10,12 CLA, (5) HFHS + 1% 10,12 CLA, or (6) HFHS + CR. CLA diets replaced 0.5% or 1% (w/w) of the lard isocalorically with 9,11 or 10,12 CLA (Nu-Chek Prep, Waterville, MN, USA >90% purity). All test diets were prepared by BioServ (Flemington, NJ, USA) in accordance with the National Research Council guidelines for rodent diets, with diet composition shown in S1 Table. Two doses (0.5 and 1%) of each CLA isomer were selected to establish an optimal dose for weight loss that appropriately mimics human CLA supplementation, based on calculations using a body surface area normalization method to compare human and mouse dose equivalencies, shown in S2 Fig [20]. CLA isomer-specific composition of each diet was confirmed using state-of-the-art NMR techniques, as shown in S3 and S4 Figs. CR was begun at 15% of baseline food intake and adjusted daily to mirror weight loss by 10,12 CLA. HFHS, 9,11 CLA, and 10,12 CLA diets were fed ad libitum. All mice were individually housed for the duration of test diet feeding. Body weights were recorded weekly. Blood was sampled at baseline and after 8 weeks of test diets (sacrifice). Mice were euthanized by exsanguination and cervical dislocation under isoflurane anesthesia, with care taken to minimize pain and discomfort throughout the study. All experimental procedures were undertaken with approval from the Institution Animal Care and Use Committee of the University of Washington (#3104–01 03/15/13–02/28/19) and followed the guidelines of the National Institutes of Health guide for the care and use of laboratory animals (NIH Publications No. 8023, revised 1978).

### Confirmation of CLA-containing diets by NMR analysis

CLA isomer-specific composition of each diet was confirmed using state-of-the-art NMR techniques. Lipid extracts of the diets containing 9,11 CLA and 10,12 CLA and the authentic standards, 9,11 CLA and 10,12 CLA, were prepared in deuterated chloroform and placed in 5mm NMR tubes for analysis. ^13^C NMR spectra were obtained, at 298 K temperature, using a Bruker AVANCE III 800MHz spectrometer equipped with a cryoprobe at ^13^C frequency of 201.21 MHz. One-dimensional NMR spectra were obtained using a one pulse sequence with a power-gated decoupling of ^1^H nuclei using WALTZ-16 pulse sequence. For each sample, 64 k data points were acquired using a spectral width of 40,000 Hz, a relaxation delay of 1 s and 64 scans. The data were processed using a spectral size of 64 k points and by multiplying with an exponential window function equivalent to a line broadening of 2.5 Hz. The resulting spectra were phase- and baseline-corrected and referenced with respect to the ^13^C signal of the chloroform solvent (77.7 ppm). Bruker Topspin version 3.0 and 3.1 software packages were used for NMR data acquisition and processing, respectively. ^13^C spectra of both diets were complex and dominated by intense peaks from lipids unconnected with 9,11 CLA and 10,12 CLA. The four olefinic carbons from each dienoic CLA, however, provided characteristic peaks in the region 126 to 136 ppm; specifically, the chemical shifts for three of the olefinic carbons were well isolated from other peaks. Initially, these peaks were tentatively assigned to 9,11 CLA or 10,12 CLA by comparing with the chemical shifts of the authentic standards. The identity of the specific CLA isomer was confirmed by spiking the lipid extracts of the diets with the authentic standards. Parts of typical ^13^C NMR spectra of the diets before and after spiking with the standards along with the spectra of the standard compounds are shown in S3 and S4 Figs.

### Body composition

Measurements of lean and fat mass were determined in conscious animals using quantitative magnetic resonance spectroscopy (QMR; EchoMRI-700TM; Echo MRI, Houston, TX, USA) at baseline (9 weeks of HFHS feeding) and after 5 weeks on test diets. Measurements were performed by the University of Washington Nutrition Obesity Research Center Energy Balance Core, as described previously [21].

### Indirect calorimetry and locomotor activity

Energy expenditure was measured using a computer-controlled indirect calorimetry system (Promethion^®^, Sable Systems, Las Vegas, NV, USA) as described previously [22]. O_2_ consumption and CO_2_ production were measured at 10-min intervals. Respiratory quotient (RQ) was calculated as the ratio of CO_2_ production to O_2_ consumption. Energy expenditure was calculated using the Weir equation [23]. Ambulatory activity was determined simultaneously with the collection of calorimetry data. Consecutive adjacent infrared beam breaks in the x-, y-, and z-axes were scored as an activity count, and a tally recorded every 10 min. Data acquisition and instrument control were coordinated by MetaScreen v.1.6.2 and raw data was processed using ExpeData v.1.4.3 (Sable Systems) using an analysis script documenting all aspects of data transformation. Mice were evaluated over three consecutive 24-hour periods, with standard alternating 12-hour light and dark cycles.

### Cold exposure

Thermogenic capacity of mice was measured during an acute and chronic cold exposure in a separate cohort of mice. Temperature transponders (Starr Life Science Corp, Oakmont, PA, USA) were implanted in the peritoneal cavity of mice after 6 weeks on test diets for continuous monitoring of core body temperature. After a 2-week recovery period, animals were acclimated to metabolic cages enclosed in temperature- and humidity-controlled cabinets (Caron Products and Services, Marietta, OH, USA). Ambient temperature was reduced from 22°C to 5°C over a 60 minute period, maintained for 6 hours (acute cold exposure), then restored to 22°C over a 60 minute period. The acute cold exposure took place after 9 weeks on test diets during the light cycle, during which mice were denied access to food. After a one-week recovery, mice were exposed to a mild chronic cold stimulus of 14°C for 72 hours. Signals emitted by transponders were sensed by a receiver positioned near the cage and analyzed using VitalView software as previously described [24]. In addition to continuously measuring core body temperature, other metabolic parameters such as locomotor activity, VȮ_2_, VcȮ_2_, heat production, and food intake (chronic cold exposure only) were measured simultaneously. For direct comparison to the shorter acute cold exposure (5°C), average 22°C exposure parameters are presented during the equivalent 6-hour time frame only.

### Glucose homeostasis

Glucose tolerance testing (intraperitoneal 1.0 mg glucose/kg lean body mass) was performed on 4-hour fasted mice after 10 weeks on HFHS diet (baseline), and after 6 weeks of test diets. Area under the curve was calculated using Graph Pad Prism software. Insulin tolerance (intraperitoneal 1 U insulin/kg lean body mass) was determined on 4-hour fasted mice after 11 weeks on HFHS diet (baseline), and again after 7 weeks of test diets.

### Plasma analyses

Plasma insulin, leptin, and adiponectin were measured using ELISAs (EMD Millipore, Temecula, CA, USA) according to the manufacturer’s instructions. Blood glucose was measured using a commercial glucometer (One Touch Ultra).

### Liver lipids and histology

Liver lipids were extracted using a Folch technique as described previously [25]. Triglycerides and cholesterol were then measured using commercially available kits from Stanbio, as described previously [26]. Glycogen content was quantified using an assay kit from Abcam. For histological analysis, formalin-fixed livers embedded in paraffin wax were sectioned at 4 μm thickness and stained with Trichrome Stain Masson Kit (Sigma-Aldrich). All stained tissue sections were visualized by Olympus BX50 microscope and then photographed using a Canon EOS 5D Mark II DSLR camera at 10X magnification. Images were analyzed using Image Pro Plus 6.0 (Media Cybernetics).

### Gene and protein expression

RNA was extracted using an RNA extraction kit (RNeasy Mini Kit, Qiagen, Valencia, CA, USA), reverse-transcribed, and cDNA amplified by qRT-PCR using an ABI 7900HT instrument. Taqman primer/probe sets for individual genes were from Thermo Fisher Scientific (Carlsbad, CA, USA). *Gapdh* was used as a reference gene. Relative expression of target genes was calculated using the ΔΔCt formula and expressed as a fold change from HFHS-fed control EWAT. For protein quantification by Western blot, 20 ug total protein from whole adipose tissue was used with PPARγ or Tubulin antibodies (Cell Signaling, Danvers, MA, USA, and Rockland Immunochemicals, Pottstown, PA, USA, respectively). For protein quantification by immunohistochemistry, formalin-fixed, paraffin-embedded adipose tissue was sectioned and stained with a monoclonal Mac2 antibody (Cedarlane Laboratories, Gymea, NSW, Canada) or a polyclonal UCP1 antibody (AbCam #ab155117, Cambridge, MA, USA) for quantification of adipose tissue macrophages and UCP1 staining, respectively, as described previously [18]. Area quantification for Mac2 and UCP1 staining was performed on digital images of immunostained tissue sections using image analysis software (Image Pro Plus software, Media Cybernetics, Rockville, MD, USA).

### Quantification of tissue fatty acids and acylcarnitines

Lipids were extracted from tissue following the Bligh and Dyer method [27]. The fatty acid components were derivatized into methyl esters as previously described [28]. Fatty acid compositions were quantified using gas chromatography (GC) using an Agilent GC machine (model 6890N) with flame ionization detector and Chemstation software for analysis.

Acylcarnitines were extracted by tissue homogenization in extraction solvent (acetonitrile and water with isotopically labeled acylcarnitine standards set B (Cambridge Isotope, Andover, MA)), and supernatants containing acyl carnitines were dried and reconstituted in 9:1 water:acetonitrile before LC-MS analysis. Agilent 1290 liquid chromatographer with Waters XBridge BEH C18 2.5um column (2.1x5cm) was used for chromatographic separation of acylcarnitines, quantified using an Agilent 6490 series triple quadrupole mass spectrometer with electrospray ionization source. Data were processed by MassHunter workstation software. Metabolite concentrations were calculated based on ratios of unlabeled metabolites and isotopically labeled metabolites and known concentrations of isotopically labeled metabolites.

### Adipocyte sizing

Adipocyte number and cross-sectional area from one randomized photomicrograph of WAT was determined using the built-in particle counting method of Image J software. Minimal particle size was set to 250 μm^2^ and circularity set to a range from 0.35 to 1.0. Individual adipocytes were assigned to arbitrary size bins ranging from 500–10,000 μm^2,^ presented as mean distribution.

### Statistics

Data were analyzed using GraphPad Prism 6 software and represented as means +/- standard error. One- and two-way ANOVA were used to compare differences between mice receiving the different diets at different time points, with Sidak or Tukey’s post-hoc tests to detect differences between groups. Calorimetry data were analyzed using a mixed linear model with a compound symmetry covariance structure [29]. A *P* value < 0.05 was considered statistically significant.

## Results

### 10,12 CLA (1%) promotes body weight reduction and subcutaneous fat loss

**Fig 1:**
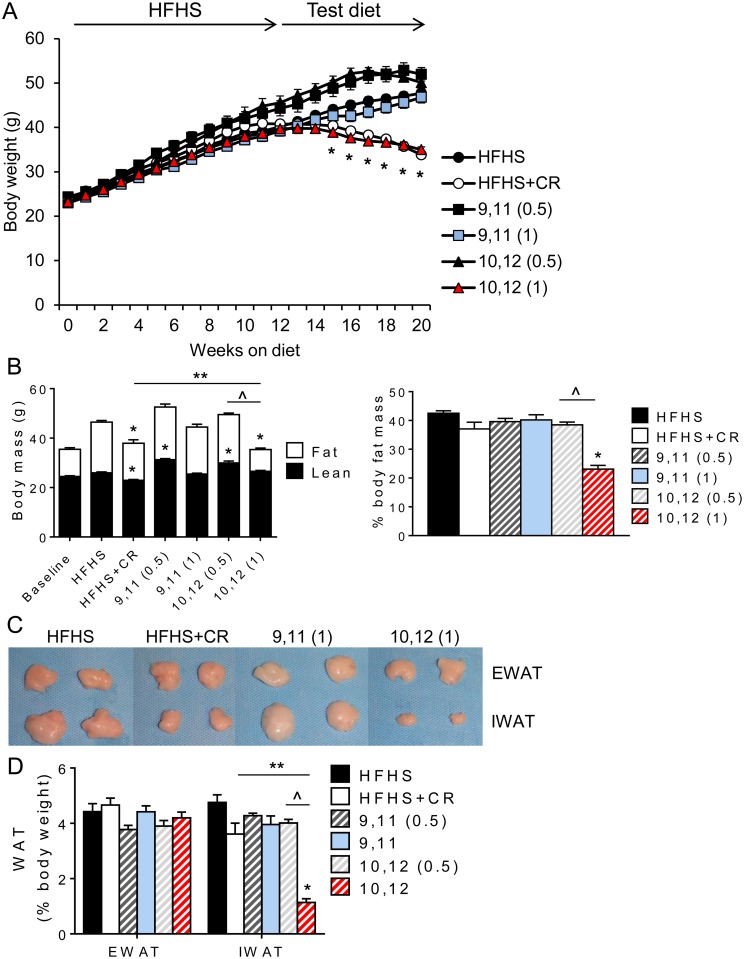
10,12 CLA (1%) promotes body weight reduction and subcutaneous fat loss. A. Body weight was measured weekly. B. Total body fat and lean mass were measured after 9 weeks (baseline) and 12+5 weeks of HFHS+test diets. Data are also presented as % body fat mass. C. Representative images of epididymal (EWAT) and inguinal (IWAT) fat pads at sacrifice. D. White adipose tissue (WAT) weights at sacrifice, expressed as percent total body weight. Data are presented as mean ± SEM, n = 10–15 mice/group. *P<0.05 from HFHS control; ^P<0.05 from 0.5% CLA; **P<0.05 from CR.

*Ldlr*^*-/-*^ mice were fed the obesogenic HFHS diet for 12 weeks, then continued on the HFHS diet with or without 9,11 or 10,12 CLA for an additional 8 weeks. All mice fed the HFHS diet gained body weight as expected, while mice fed 1% 10,12 CLA lost significant body weight (Fig 1A). In contrast, mice fed 0.5 or 1% 9,11 CLA or 0.5% 10,12 CLA failed to lose body weight, supporting evidence that 10,12 CLA is responsible for CLA-induced weight loss and that the 1% dose was most effective in this model. To control for effects due simply to weight loss, an additional group of mice underwent CR to mirror the weight loss by 1% 10,12 CLA. 1% 10,12 CLA-induced weight loss was due to the loss of body fat and percent body fat mass, with lean body mass maintained (Fig 1B). In contrast, CR mice exhibited reductions in both body fat and lean body mass. Surprisingly, following dissection of individual fat pads (Fig 1C), the percentage of visceral fat (EWAT) was maintained by 1% 10,12 CLA, while subcutaneous fat storage (IWAT) was dramatically reduced (Fig 1D). It is important to note that IWAT mass was largely maintained with CR as a percentage of total body fat. Since the 0.5% CLA-containing diets did not induce changes in body metrics or any subsequent energy balance or tissue measurements, only data from the 1% CLA groups will be shown hereafter. Collectively, these data demonstrate that 1% 10,12 CLA supplementation causes weight loss by reducing fat mass and protecting against the loss of lean body mass, due to a select reduction in subcutaneous fat with preservation of visceral adipose tissue.

### Energy expenditure and respiratory quotient are increased by 10,12 CLA

**Fig 2:**
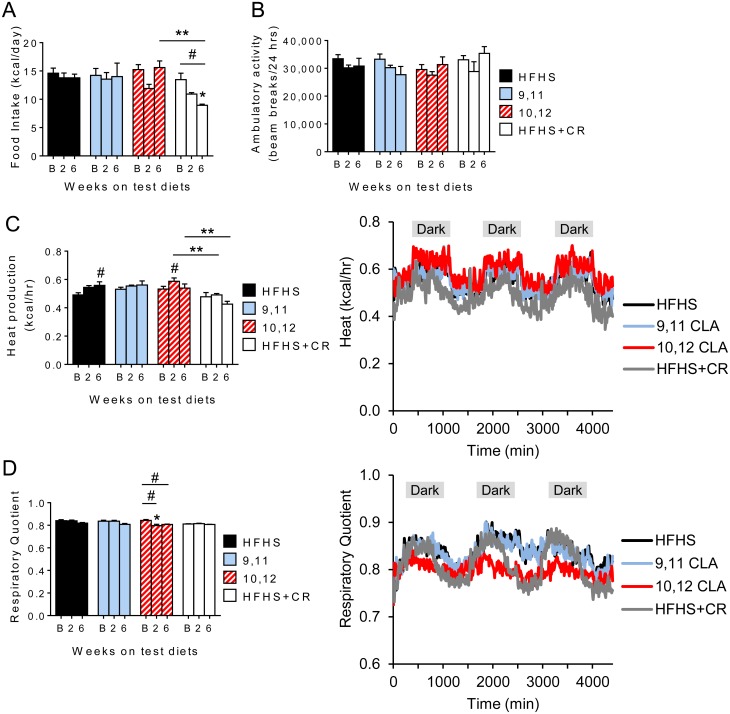
Energy expenditure and respiratory quotient are increased by 10,12 CLA. A. Food intake, B. ambulatory activity, C. heat production, and D. respiratory quotient were measured every 10 minutes for 3 days at baseline (B: 12 weeks on HFHS diet), and again 2- and 6-weeks after initiation of the indicated diets, and presented as the average values. Representations of continuous heat production (C) and respiratory quotient (D) measurements are indicated. Shaded areas indicate the dark cycle. Data are presented as mean ± SEM, n = 8-12/group. ^#^P<0.05 from baseline, *P<0.05 from HFHS control, **P<0.05 from CR.

We next determined whether 10,12 CLA-mediated weight loss was due to changes in energy intake, energy expenditure or both. Food intake did not differ significantly between groups, with the intentional exception of the CR group (Fig 2A). Energy expenditure, respiratory quotient, and ambulatory activity were measured at baseline (B, measured after 11 weeks of HFHS feeding), and again at 2 and 6 weeks after introduction of the test diets. While ambulatory activity was unchanged (Fig 2B), energy expenditure was increased by 10,12 CLA at 2 weeks, prior to changes in body weight (Fig 2C). Further, while CR mice reduced energy expenditure after 6 weeks, consistent with that observed in humans [30], this was maintained in 10,12 CLA-treated animals. This maintenance of energy expenditure during negative energy balance is likely due to the protection of lean body mass stores, since this effect is abolished when energy expenditure is normalized to lean body mass (S5A–S5C Fig). Moreover, using a linear mixed model approach as recommended for the analysis of energy expenditure [29], our results further indicate that 10,12 CLA-treated mice exhibited higher body mass-adjusted energy expenditure compared to control groups (S5D–S5F Fig). In addition, RQ was decreased by 10,12 CLA only, indicative of increased fat oxidation (Fig 2D). Examples of the cumulative heat production and RQ measurements taken after 2 weeks of test diets show that these parameters remained elevated and suppressed, respectively, in 10,12 CLA-treated mice for the duration of measurements (Fig 2C and 2D). Taken together, these data suggest that 10,12 CLA induces weight loss by increasing energy expenditure and fat oxidation, without altering energy intake or locomotor activity.

### 10,12 CLA fails to improve glucose metabolism

**Fig 3:**
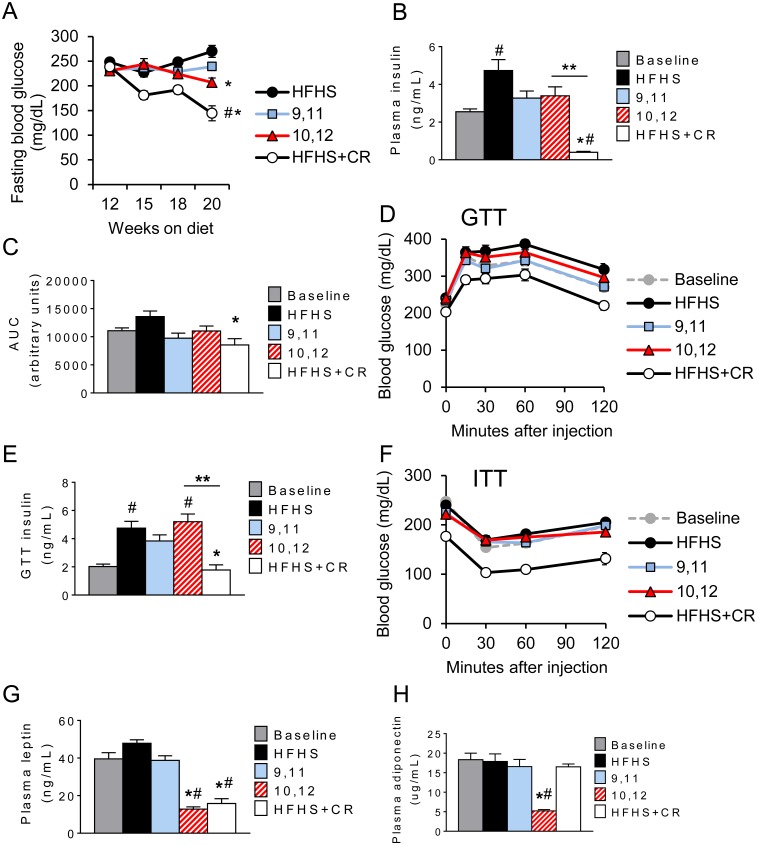
10,12 CLA fails to improve glucose metabolism. A. Fasting blood glucose taken at indicated time points. B. Fasting plasma insulin measured at sacrifice. C-D. Area under the curve (AUC, C) for glucose tolerance test (GTT, D) performed at 10 weeks (baseline) and 12+6 weeks on HFHS+indicated diets. E. Plasma insulin measured at the 30 minute time point during the GTT. F. Insulin tolerance test (ITT) performed at 11 weeks (baseline) and 12+7 weeks on the indicated diets. G-H. Fasting plasma leptin (G) and adiponectin (H) measured at baseline and sacrifice. Data are presented as mean ± SEM, n = 10-15/group. ^#^P<0.05 from baseline, *P<0.05 from HFHS control, **P<0.05 from CR.

Weight loss in rodent and human studies is well characterized to improve glucose metabolism [31]. Consistent with this, we found that CR reduced fasting blood glucose and plasma insulin levels compared with baseline levels taken prior to initiation of the test diets (Fig 3A and 3B). However, despite similar weight loss, these improvements were not observed in 10,12 CLA-treated animals. To examine this further, mice were subjected to a glucose tolerance test (GTT, Fig 3C and 3D). Again, CR mice exhibited a marked improvement in glucose tolerance, an effect that could not be explained by increased insulin secretion, as plasma insulin levels at 30 minutes during the GTT were reduced (Fig 3E). In contrast, glucose tolerance was not improved by 10,12 CLA, with no evidence of an insulin secretory defect (Fig 3E). To examine the effects on insulin sensitivity, we next subjected mice to an insulin tolerance test (ITT, Fig 3F). Again, insulin tolerance was improved by CR, but not by 10,12 CLA. Consistent with the loss in body fat, plasma leptin was significantly reduced by both CR and 10,12 CLA (Fig 3G). However the insulin-sensitizing adipokine adiponectin was decreased by 10,12 CLA, but importantly was not decreased by CR (Fig 3H). Collectively, these data suggest that despite weight loss, 10,12 CLA fails to improve glucose metabolism unlike CR, an effect that may be partially explained by the preferential loss of subcutaneous fat and subsequent reductions in plasma adiponectin.

### 10,12 CLA worsens hepatic triglyceride content

**Fig 4:**
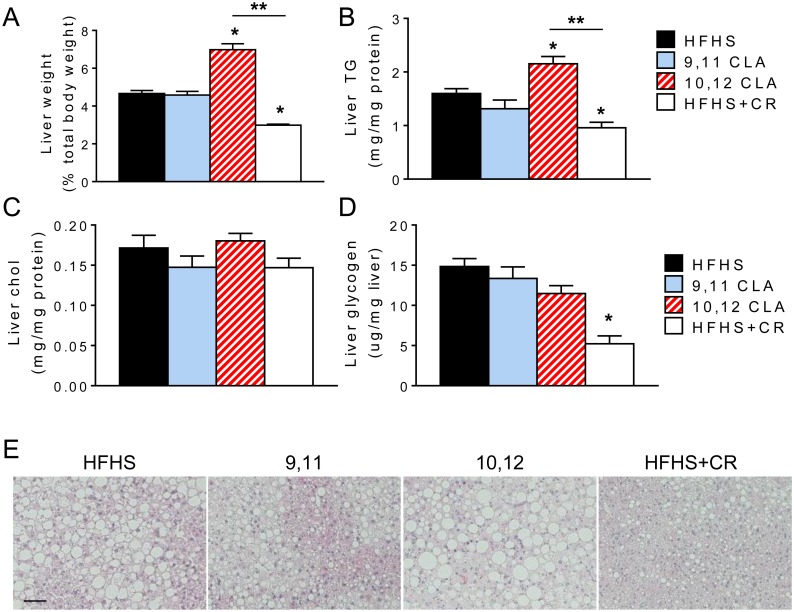
10,12 CLA worsens hepatic triglyceride content. A. Liver weight expressed as a percentage of total body weight. B-D. Liver triglycerides (TG, B), cholesterol (C) and glycogen (D) were measured, normalized to total protein or liver weight. E. Representative images of liver sections stained with hematoxylin and eosin, scale bar = 400 μm. Data are presented as mean ± SEM, n = 5-15/group. *P<0.05 from HFHS control, **P<0.05 from CR.

Given its prominent role in whole-body glucose regulation [32], we next examined the livers from these mice. Liver weight was increased by 10,12 CLA, which can be attributed to increased hepatic triglyceride content, but not cholesterol or glycogen (Fig 4A–4D). This was evident in images of hematoxylin- and eosin-stained liver sections (Fig 4E), in which hepatic steatosis is more pronounced in 10,12 CLA-supplemented mice. Conversely, liver weight, triglycerides, glycogen, and hepatic steatosis were decreased by CR. We next measured expression levels of genes involved in lipid metabolism, glucose metabolism, and inflammation (Table 1). 10,12 CLA did not alter expression of genes involved in fatty acid synthesis (fatty acid synthase (*Fasn*) and acetyl coA carboxylase (*ACC*)), sterol synthesis (sterol regulatory element-binding transcription protein 1c (*Srebp1c*)), triglyceride synthesis (diglycerol acyltransferase 1 (*Dgat1*)), fatty acid oxidation (carnitine palmitoyl transferase 1a (*Cpt1a*)), or gluconeogenesis (phosphoenolpyruvate carboxykinase (*Pepck*) and glucose 6 phosphatase (*G6pc3*)), but increased expression of solute carrier family 2 member 2 (*Slc2a2*), the gene that encodes GLUT2, which facilitates insulin-independent glucose uptake by the liver. Moreover, 10,12 CLA increased expression of inflammatory genes interleukin 6 (*Il6*) and chemokine (C-C motif) ligand 2 (*Ccl2*). However, CR led to decreased expression of hepatic fatty acid and cholesterol synthesis genes (*Fasn* and *Srebp1c*, respectively), increased expression of the fatty acid oxidation gene *Cpt1a*, and increased the gluconeogenesis gene *Pck1*. Taken together, these results suggest that 10,12 CLA worsens hepatic steatosis by increasing triglyceride storage and inflammation in the liver, while CR improves hepatic steatosis by simultaneously reducing fatty acid synthesis and cholesterol synthesis gene programs while increasing fatty acid oxidation.

**Table 1:** Liver gene expression.

Gene	HFHS	9,11	10,12	HFHS+CR
1. *Fasn*	1.00±0.11	0.88±0.09	0.90±0.08	0.38±0.05*
2. *Acc*	1.00±0.09	1.02±0.14	1.19±0.11	0.88±0.12
3. *Srebp1c*	1.00±0.12	0.87±0.09	0.68±0.11	0.32±0.04*
4. *Dgat1*	1.00±0.19	1.06±0.16	1.29±0.17	1.92±0.49
5. *Cpt1a*	1.00±0.09	1.14±0.11	1.13±0.11	2.29±0.16*
6. *Pck1*	1.00±0.14	1.09±0.15	1.16±0.29	2.59±0.37*
7. *G6pc3*	1.00±0.03	1.04±0.09	1.12±0.10	0.92±0.07
8. *Slc2a2*	1.00±0.05	1.23±0.06	1.39±0.15*	1.35±0.07*
9. *Il6*	1.00±0.22	1.16±0.21	2.11±0.44*	1.52±0.22
10. *Ccl2*	1.00±0.23	1.13±0.14	1.73±0.23*	0.59±0.08

Expression levels of lipid metabolism (1–5), glucose metabolism (6–8), and inflammatory (9–10) genes from liver, expressed as a fold change from the HFHS control group ± SEM.

*P<0.05 from HFHS control.

### Differential expression of lipid metabolism genes in visceral and subcutaneous depots in response to 10,12 CLA and caloric restriction

**Fig 5:**
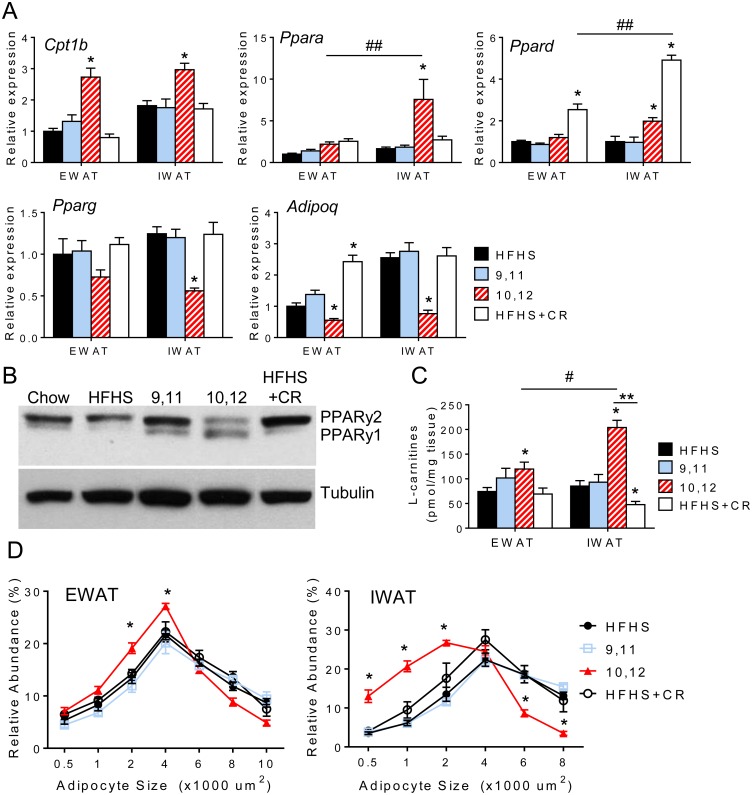
Differential expression of lipid metabolism genes in visceral and subcutaneous depots in response to 10,12 CLA and caloric restriction. A. Expression of lipid catabolism genes (*Cpt1b*, *Ppara*, *Ppard*) and adipogenic genes (*Pparg* and *Adipoq*) from EWAT and IWAT (normalized to HFHS-fed EWAT). B. Representative Western blot for PPARγ, with tubulin used as a loading control. C. L-carnitine levels in EWAT and IWAT. D. Adipocyte size distribution of EWAT (left) and IWAT (right). Data are presented as mean ± SEM, n = 10-15/group. *P<0.05 from HFHS control, ^#^P<0.05 from EWAT, **P<0.05 from CR.

To gain insights into the mechanism(s) whereby 10,12 CLA mediates its effects on energy expenditure to induce fat loss, we measured expression of key genes regulating lipid metabolism in WAT. Consistent with a reduction in RQ, we found that *Cpt1b*, a gene essential for adipose tissue fatty acid oxidation (FAO), was significantly upregulated by 10,12 CLA in both EWAT and IWAT, while expression of its upstream transcription factors *Pparα* and *Pparδ* were increased in IWAT only (Fig 5A). Further, the expression of adipogenic genes such as *Pparγ* and its downstream target *Adipoq* were significantly reduced by 10,12 CLA in IWAT and to a lesser degree in EWAT, with confirmed reduction in PPARγ2 protein, but increased PPARγ1 protein (Fig 5B). To determine if enhanced lipid catabolism altered tissue fatty acids, we quantified specific and total fatty acids from EWAT and IWAT, but did not detect any diet-specific differences (S6A Fig). Fatty acids can only enter the mitochondria to undergo FAO after they have been esterified into acyl-CoA, followed by conversion into acylcarnitines by CPT1. Therefore, acylcarnitines are a good measure for estimating FAO levels. Total L-carnitines were significantly increased by 10,12 CLA in IWAT (Fig 5C), and individual acylcarnitines separated using LC-MS were similarly enriched in IWAT, but not liver (S6B Fig). In contrast, acylcarnitines were reduced in IWAT after CR. Finally, the adipocyte size distribution in EWAT and to a greater extent in IWAT was left-shifted towards an abundance of smaller adipocytes in 10,12 CLA-treated mice (Fig 5D), but not in CR mice. Taken together, these data suggest that 10,12 CLA skews the balance away from a traditional lipid storage program towards lipid catabolism, and IWAT appears to be more responsive to this switch than EWAT.

### Inguinal white adipose tissue becomes enriched with alternatively activated macrophages following 10,12 CLA supplementation

**Fig 6:**
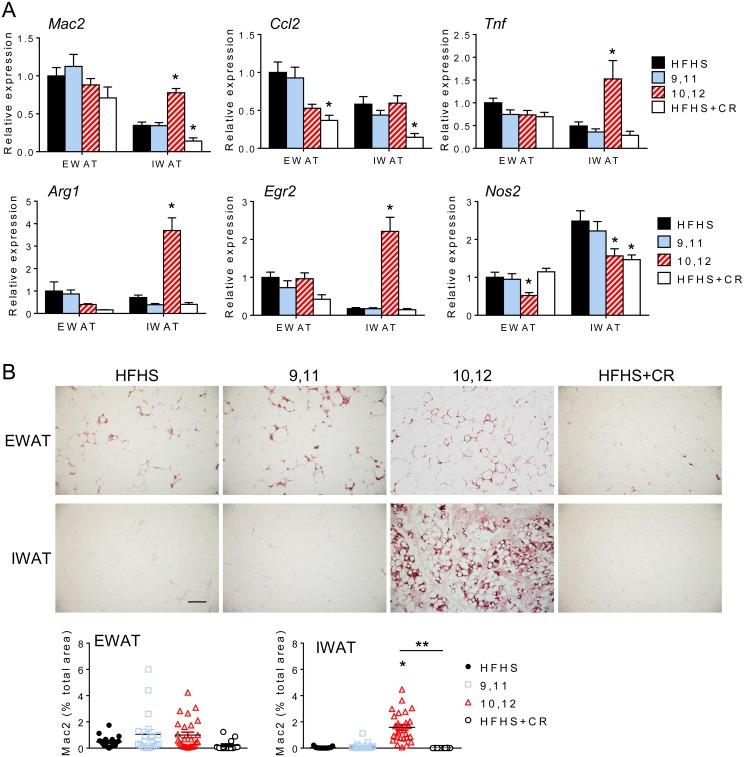
Inguinal white adipose tissue becomes enriched with alternatively activated macrophages following 10,12 CLA supplementation. A. Expression of genes representative of macrophages (*Mac2*), chemotactic factors (*Ccl2*), inflammation (*Tnf*), alternatively activated macrophages (*Arg1*, *Egr2*), and pro-inflammatory macrophages (*Nos2*) from EWAT and IWAT (normalized to HFHS-fed EWAT). B. Representative images of Mac2 immunostaining from EWAT and IWAT, scale bar = 300 μm. Percent total Mac2 stained area for EWAT (left) and IWAT (right) shown below. Data are presented as mean ± SEM, n = 10-15/group. *P<0.05 from HFHS control, **P<0.05 from CR.

Weight loss has been associated with improved WAT inflammation and leukocyte content [33]. Consistent with this, CR promoted reductions in *Mac2*, a global macrophage marker, the macrophage chemotactic factor *Ccl2*, and the pro-inflammatory cytokine tumor necrosis factor *(Tnf)* (Fig 6A). However, 10,12 CLA promoted increased expression of *Mac2* in subcutaneous IWAT (Fig 6A). Macrophages in 10,12 CLA-supplemented mice can be further characterized as alternatively activated, or “M2”, macrophages, exhibiting increased expression of Arginase 1 (*Arg1*) and early growth response protein 2 (*Egr2*) with decreased expression of inducible nitric oxide (*Nos2*), a classic “M1” inflammatory macrophage marker (Fig 6A) [34]. Immunohistochemistry confirmed that Mac2 protein levels were increased by 10,12 CLA in IWAT only (Fig 6B). These data suggest that inflammatory gene expression and alternatively activated macrophages are elevated by 10,12 CLA in subcutaneous WAT.

### 10,12 CLA induces browning of white adipose tissue

**Fig 7:**
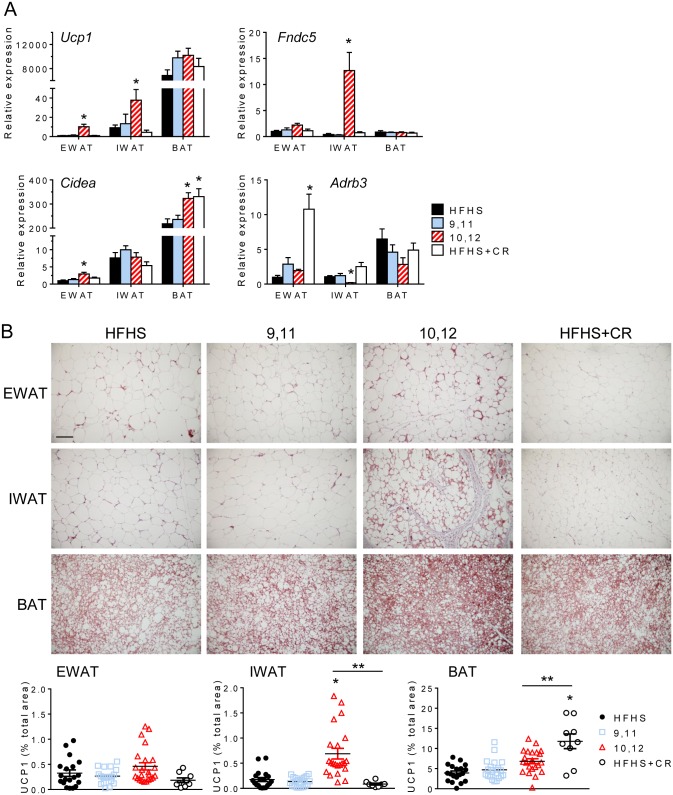
10,12 CLA induces the browning of white adipose tissue. A. Expression of genes associated with browning (*Ucp1*, *Fndc5*, *Cidea*, *Adrb3*) from EWAT, IWAT, and BAT (normalized to HFHS-fed EWAT). B. Representative images of UCP1 immunostaining from EWAT, IWAT, and BAT, scale bar = 300 μm. Percent total UCP1 stained area for EWAT (left), IWAT (middle), and BAT (right) shown below. Data are presented as mean ± SEM, n = 5-15/group. *P<0.05 from HFHS control, **P<0.05 from CR.

Given the increase in energy expenditure, we examined whether 10,12 CLA activates brown adipose tissue (BAT). Interscapular BAT FAO was increased equally in response to 10,12 CLA and CR, likely attributable to weight loss as both groups also lost BAT mass (S7A Fig). BAT expression of *Ucp1* and *Cidea* was unchanged by 10,12 CLA (Fig 7A), suggesting that subscapular BAT is not activated above basal levels.

The browning of WAT, in which UCP1-positive cells are present within WAT depots, has recently been described as a potential mechanism for fat loss [reviewed by [35]], and can occur in response to physiological or pharmacological stimuli. With depot-specific alterations in FAO, a preferential metabolic pathway of brown adipocytes, we sought to determine if 10,12 CLA induces the browning of WAT. *Ucp1* and *Cidea* gene expression was increased in both EWAT and IWAT (Fig 7A), with higher UCP1 mRNA and protein detected in IWAT from 10,12 CLA-supplemented mice (Fig 7B). Expression of beta-3 adrenergic receptors (*Adrb3*), the primary efferent receptors linked to the sympathetic control of browning, were unchanged or decreased by 10,12 CLA in EWAT and IWAT, respectively (Fig 7A), suggesting that this browning is not sympathetically driven. Irisin, a recently identified myokine that has known WAT browning effects, was not altered at the transcriptional level in skeletal muscle or in the plasma by any treatment (S7B Fig), suggesting that 10,12 CLA does not mediate its browning effects through muscle-derived irisin. However, expression of *Fndc5*, the gene that encodes irisin, was significantly elevated by 10,12 CLA in IWAT but not EWAT or BAT, providing a potential autocrine/paracrine mechanism of browning in this WAT depot. Taken together, these data suggest that while 10,12 CLA does not appear to activate interscapular BAT, it does promote the browning of WAT by a mechanism potentially involving locally produced irisin that may be independent from the classic sympathetically-driven pathway.

### Cold exposure potentiates 10,12 CLA-induced increases in energy expenditure

**Fig 8:**
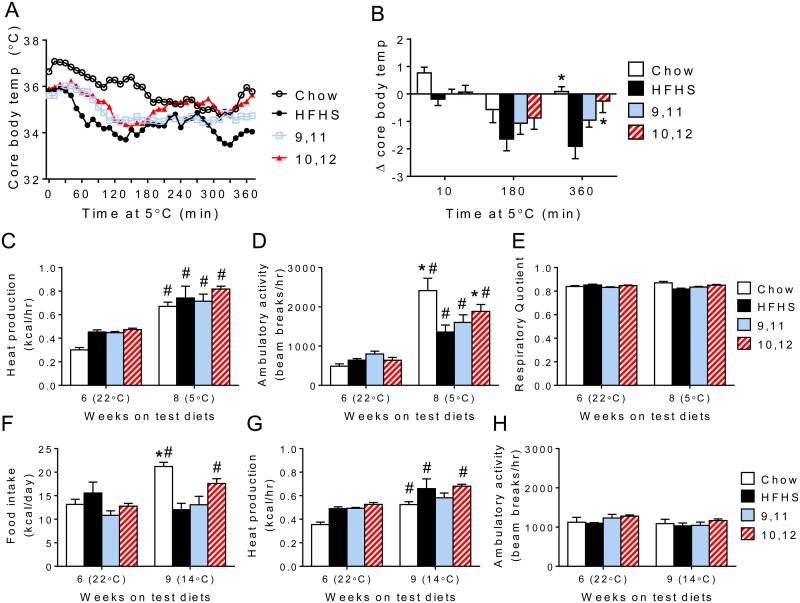
Cold exposure potentiates 10,12 CLA-induced increases in energy expenditure. A. After 12+9 weeks on HFHS+indicated diets, a separate group of mice were housed at 5°C for 6 hours and core body temperatures recorded every 10 minutes. B. The change from time = 0 was calculated after 10, 180, and 360 minutes of cold exposure. C-E. Heat production (C), locomotor activity (D) and respiratory quotient (RQ) (E) were measured continuously during an ambient room temperature exposure (22°C for 72 hours, after 8 weeks on test diets) followed by an acute cold exposure (5°C for 6 hours after 9 weeks on test diets). 22°C data in (C-E) are adjusted to reflect only values collected during equivalent time periods as the 5°C exposure. F-H. Food intake (F), heat production (G), and ambulatory activity (H) were measured during the same ambient room temperature exposure (22°C for 72 hours, after 8 weeks on test diets) and again during a chronic moderately cold exposure (14°C for 72 hours, after 10 weeks on test diets). Data are presented as mean ± SEM, n = 4/group. ^#^P<0.05 from 22°C, *P<0.05 from HFHS control.

Based on the increased energy expenditure and WAT browning by 10,12 CLA, we determined whether these mice exhibited improvements in cold-induced thermogenesis. No differences in core body temperature were observed between groups at normal ambient temperature (22°C, S8A Fig), so we examined mice following (1) an acute cold exposure (5°C for 6 hours) and (2) a chronic mild cold exposure (14°C for 72 hours) relative to lean chow-fed controls. After an initial dip in core body temperature, 10,12 CLA-treated mice were able to recover from the acute cold exposure similarly to chow-fed mice (Fig 8A and 8B). This was in contrast to HFHS-fed mice that showed a continuous decline in core body temperature. As expected, all mice exhibited increased heat production during the acute cold exposure, which could be partially attributed to increased locomotor activity in the chow and 10,12 CLA groups (Fig 8C and 8D). Moreover, again using a linear mixed model approach, our results indicate that 10,12 CLA-treated mice exhibited significantly higher body mass-adjusted energy expenditure compared to control groups during both cold exposures (S8B–S8D Fig). RQ was unchanged by cold exposure (Fig 8E). As expected, chronic mild cold exposure (14°C) caused chow-fed animals to increase their food consumption, but this was not observed in the HFHS diet-based groups (Fig 8F), while all groups showed similar increases in heat production, an effect that could not be explained by increased locomotor activity (Fig 8G and 8H).

## Discussion

While supplements containing 10,12 CLA are commonly used for weight loss, their efficacy, safety, and mechanism of action have not been conclusively determined. In this study we examined 10,12 CLA supplementation in obese mice with characteristics of human MetS with direct comparison with weight-matched CR mice. Previous studies suggest that higher 10,12 CLA doses that promote greater weight loss in humans are associated with adverse effects such as systemic inflammation and insulin resistance [16, 36]. The CLA dose used in this study is well within the range of human CLA supplementation, confirming that our mouse model of MetS approximates human supplementation. We now report that 1% 10,12 CLA causes weight loss by reducing subcutaneous fat mass without affecting visceral epididymal fat stores, while 9,11 CLA has relatively inert effects. This weight loss could not be explained by changes in food intake or locomotion, but by increased energy expenditure and reduced RQ, with accompanying increases in FAO and selective browning of subcutaneous WAT. However, relative to equivalent weight loss induced by CR, 10,12 CLA-induced weight loss failed to improve glucose metabolism, an effect attributed to excess visceral fat and inflammation, reduced subcutaneous fat and circulating adiponectin, and worsened hepatic steatosis. Taken together, our findings provide novel insights into the mechanism(s) whereby 10,12 CLA promotes weight loss without the expected improvements in insulin sensitivity, enabled by direct comparison with a weight matched CR control group.

While it is known that 10,12 CLA reduces body weight and adiposity, we have shown for the first time a distinct adipose depot-specific effect in mice presenting with a humanoid MetS phenotype. Our findings demonstrate the selective reduction in subcutaneous WAT with 10,12 CLA that is characterized by increased FAO, higher levels of acylcarnitine species, smaller adipocytes, increased inflammation and macrophages, and decreased adiponectin levels. While CR reduced hepatic steatosis by reducing fatty acid synthesis and promoting fatty acid oxidation, our group and others have now shown that 10,12 CLA worsens hepatic steatosis [37, 38], an effect that is associated with insulin resistance [39]. Collectively, these liver and IWAT characteristics likely explain the failure to improve glucose metabolism relative to CR animals despite similar weight loss. While smaller adipocytes are known to secrete higher levels of adiponectin [40], inflammatory cytokines blunt adiponectin production [41]. Subcutaneous adipocytes were smaller in response to 10,12 CLA, but they were also more inflamed, which could have impacted circulating adiponectin levels. Consistent with our observations, visceral WAT volume is more strongly correlated with metabolic risk factors such as insulin resistance than subcutaneous mass [42]. Further, studies in humans suggest that subcutaneous WAT contributes more to circulating adiponectin levels [43]. As adiponectin is known to have insulin-sensitizing properties [44], it is likely that 10,12 CLA-induced subcutaneous fat loss contributes to reduced plasma adiponectin and subsequent insulin resistance. Taken together, the lack of improvement in glucose metabolism seen with 10,12 CLA supplementation is likely due, in part, to the loss of subcutaneous WAT and associated loss of adiponectin, with an increased proportion of visceral WAT.

While subcutaneous fat catabolism is promoted by 10,12 CLA, adipogenic programs appear to be compromised. We have previously shown that expression of *Pparg* and its downstream targets *Adipoq* and *Hsl* are reduced by 10,12 CLA in cultured adipocytes, with reduced triglyceride storage [13]. We now show that *Pparg* and *Adipoq* gene expression and PPARγ2 protein expression are significantly reduced by 10,12 CLA in IWAT. There is emerging evidence that PPARγ preferentially directs lipid storage to subcutaneous adipose tissue, as the PPARγ agonist rosiglitazone induces 2-fold more subcutaneous triglyceride storage than visceral fat storage [45]. The lipid storage and insulin-sensitizing effects of PPARγ are largely attributed to the PPARγ2 isoform [46], which we show is reduced by 10,12 CLA. Conversely, we show that PPARγ1 protein is increased by 10,12 CLA, although this more ubiquitous PPARγ isoform is not as important for adipocyte lipogenesis as PPARγ2 [47]. In addition, perilipin has been shown to decrease in WAT in response to CLA [48], suggesting that a defect in lipid storage promotes the oxidation of otherwise toxic lipid accumulation. Moreover, moderate weight loss increases circulating adiponectin [49]. Adiponectin levels were maintained by CR, but surprisingly adiponectin *decreased* with 10,12 CLA-associated weight loss. The loss of subcutaneous WAT evoked by 10,12 CLA is similar to the effects of human partial lipodystrophy, in which peripheral adipose tissue is redistributed to visceral adipose depots or ectopic sites such as the liver, and is often linked with defective PPARγ signaling and insulin resistance [50]. We speculate that the loss of the adipogenic program in subcutaneous WAT drives the phenotypic switch towards FAO, facilitating adipocyte shrinkage which retains inflammatory macrophages and promotes insulin resistance by ectopic deposition of triglyceride in the liver. While the novelty of this study was the direct comparison with a weight-matched CR control group, a future direction would be to include a body fat-matched control group by utilizing intermittent CR, which promotes the loss of body fat while maintaining lean body mass [51].

Decreased PPARγ signaling, suggestive of reduced adipogenesis that is consistent with the loss of subcutaneous WAT, does not complement our observed increases in the browning of subcutaneous WAT. It has been established that both white and brown adipocytes require intact PPARγ signaling for differentiation [52, 53]. However, Spiegelman et al. have recently described a novel pathway by which browning of WAT occurs that is driven by PPARα [54]. Irisin, a myokine released in response to skeletal muscle PGC1α overexpression or exercise, promotes the browning of subcutaneous WAT with simultaneous reductions in WAT adiponectin expression [54], suggesting a PPARγ-independent mechanism. While we did not detect any changes in skeletal muscle *Fndc5* expression or plasma irisin, we did observe increased *Fndc5* expression in IWAT, suggesting that irisin could exert autocrine or paracrine effects on WAT. PPARα is capable of driving UCP1 expression in adipocytes [55], potentially mediated by irisin [56]. 10,12 CLA clearly induces the expression and activity of subcutaneous WAT PPARα and irisin, which raises the intriguing possibility that the browning of WAT in response to 10,12 CLA could be PPARα- and/or irisin-mediated. Moreover, a role for alternatively activated macrophages in the browning of white adipose tissue is becoming increasingly recognized. Nguyen et al. and Qiu et al. recently showed that alternatively activated macrophages provide a critical source of norepinephrine, the major sympathetic neurotransmitter that causes WAT browning [57, 58]. Coupled with our observed reduction in *Adrb3*, the principal beta adrenergic receptor that promotes browning mediated by the sympathetic nervous system (SNS), it is possible that 10,12 CLA promotes WAT browning by recruitment of alternatively activated macrophages independently of the SNS. Pini et al. recently showed that lean mice given 10,12 CLA by oral gavage for 7 days had enriched M2 macrophage populations in both gonadal and inguinal WAT [59], suggesting that the accumulation of M2 macrophages in response to 10,12 CLA is not exclusive to the obese state. Future studies that perturb adipose tissue macrophages will determine a role of alternatively activated macrophages in the WAT browning response to 10,12 CLA.

That 10,12 CLA increases basal metabolic rate has been suggested previously in mice and humans [60, 61], in which mixed CLA isomers were administered for 5 weeks (mice) and 13 weeks (humans), although subjects in those studies did not lose weight or gain lean mass. In our study mice fed only 10,12 CLA showed an increase in energy expenditure that was enhanced when adjusted for total body mass, in addition to significant total weight loss. This is in striking contrast to CR mice that exhibited a marked reduction in energy expenditure with similar weight loss. Notably, when normalized to lean body mass, the increased energy expenditure by 10,12 CLA was abolished, suggesting that the lean compartment contributes to this effect. Moreover, the reduced RQ observed with 10,12 CLA supports our observed increase in adipose tissue *Cpt1β* expression, suggesting the metabolic substrate preference for fat in these mice.

Our studies confirm and extend previous reports that 10,12 CLA is associated with browning of WAT [11, 62]. Moreover, 10,12 CLA failed to activate BAT (assessed by *Ucp1* mRNA and protein levels), also consistent with previous findings [62]. In contrast to previous work by Shen et al. [11, 62], our study has confirmed that 10,12 CLA modulates the browning of WAT in an obese mouse model with features of MetS, which more closely resembles an unhealthy human population that would actively seek weight loss compounds such as CLA. Further, we show that in this MetS model of 10,12 CLA-mediated weight loss, WAT browning occurs exclusively in subcutaneous WAT coincidently with elevated FAO and alternatively activated macrophages. While we do see a small increase in WAT UCP1 mRNA and protein expression in EWAT, as has previously been reported [62, 63], most of the WAT browning effect in our model is exclusive to IWAT. While additional studies are required to determine whether 10,12 CLA-mediated WAT browning is mediated by the SNS, we did not detect an increase in WAT β_3_-adrenergic receptor expression. Non-sympathetically driven mechanisms of WAT browning would have therapeutic potential, as previous attempts to promote browning using beta adrenergic agonists have had undesirable cardiometabolic side effects [64]. However, it is still not clear the extent to which browning of WAT contributes to 10,12 CLA-induced weight loss or thermoregulation. Nonetheless, 10,12 CLA-supplemented mice maintained their core body temperature akin to lean chow-fed controls during a cold challenge, an effect associated with an increase in locomotor activity in both chow- and 10,12 CLA-fed mice. However, during chronic mild cold exposure, where animals rely primarily on adaptive thermogenesis to generate heat [65], heat production was elevated despite no changes in ambulatory activity.

In summary, we have shown that mice with characteristics of human MetS lose significant body weight with 10,12 CLA supplementation, largely due to the loss of subcutaneous but not visceral fat. This loss of subcutaneous fat is coupled with localized macrophage accumulation, reduced adipokines, and worsened hepatic steatosis, all of which likely contribute to the failure to improve glucose metabolism. Moreover, this 10,12 CLA-induced weight loss is due to increased energy expenditure, rather than changes in food intake, an effect that may occur, in part, due to a relative increase in lean body mass and/or the browning of WAT. These findings are in stark contrast to the metabolic effects in weight-matched calorically restricted mice, which maintained subcutaneous fat stores and adiponectin levels while decreasing hepatic lipid storage, and exhibited the expected improvements in glucose metabolism. These results provide new evidence that 10,12 CLA exerts profound effects on adiposity by redistributing fat stores away from healthy subcutaneous regions. This study suggests that weight loss achieved by 10,12 CLA is not metabolically “healthy”, but with additional co-therapies aimed at improving insulin sensitivity, 10,12 CLA as a weight loss supplement could still be useful.

## Supporting information

S1 Fig Study schematic. Two separate cohorts of male *Ldlr*^-/-^ mice were fed a HFHS diet for 12 weeks, then continued on the HFHS diet with or without the addition of 0.5% 9,11 CLA, 1% 9,11 CLA, 0.5% 10,12 CLA, 1% 10,12 CLA, or 15% CR for an additional 8–10 weeks. Mice in cohort 1 underwent body composition, glucose tolerance testing, insulin tolerance testing, and indirect calorimetry as indicated on the timeline prior to sacrifice after 8 weeks on test diets (n = 10–15 mice/group). Mice in cohort 2 were implanted with a temperature transponder after 6 weeks on test diets. Following a 2-week recovery, indirect calorimetry was measured continuously during exposures to three separate ambient temperatures: (1) normal ambient room temperature (22°C for 3 days), (2) an acute cold challenge (5°C for 6 hours), and (3) a chronic cold challenge (14°C for 3 days) (n = 4 mice/group). A group of lean *Ldlr*^-/-^ mice consuming normal rodent chow were used as a control group in cohort 2.
(TIF)

S2 Fig Body surface area normalization of CLA doses. Representative calculations using a body surface area normalization method (Reagan-Shaw et al., [20]) to compare human and mouse CLA dose equivalencies.
(TIF)

S3 Fig Confirmation of the 9,11 CLA diet. Parts of ^13^C NMR spectra of (a) 9,11 CLA diet; (b) 9,11 CLA diet spiked with 9,11 CLA standard; (c) 9,11 CLA diet spiked with 10,12 CLA standard; (d) 9,11 CLA standard; and (e) 10, 12 CLA standard. ^13^C chemical shifts of the four olefinic carbons were used to identify the specific CLA, unambiguously. The spectra were obtained on a Bruker Avance III 800 MHz spectrometer.
(TIF)

S4 Fig Confirmation of the 10,12 CLA diet. Parts of ^13^C NMR spectra of (a) 10,12 CLA diet; (b) 10,12 CLA diet spiked with 10,12 CLA standard; (c) 10,12 CLA diet spiked with 9,11 CLA standard; (d) 9,11 CLA standard; and (e) 10, 12 CLA standard. ^13^C chemical shifts of the four olefinic carbons were used to identify the specific CLA, unambiguously. The spectra were obtained on a Bruker Avance III 800 MHz spectrometer.
(TIF)

S5 Fig Lean body mass- and total body mass-adjusted calorimetry. Indirect calorimetry was performed as described in the experimental procedures section at baseline (B), 2 weeks, and 6 weeks after the introduction of the test diets. A. Lean body mass-adjusted oxygen consumption (VȮ_2_), B. carbon dioxide production (VCȮ_2_), and C. heat production (kcal/kg/hour). D. Total body mass-adjusted oxygen consumption, E. carbon dioxide production, and F heat production (kcal/kg lean/hour). Data are presented as mean ± SEM, n = 8-12/group. Data were analyzed using a mixed linear model with a compound symmetry covariance structure. *P<0.05 from HFHS control, **P<0.05 from CR.
(TIF)

S6 Fig Tissue fatty acids and acylcarnitines. A. Selected fatty acid composition of epididymal white adipose tissue (EWAT, left) or inguinal white adipose tissue (IWAT, right) from mice fed the indicated diets. B. Selected acylcarnitine species of EWAT, IWAT, or liver from mice fed the indicated diets. Data are presented as mean ± SEM, n = 5/group.
(TIF)

S7 Fig BAT characteristics and irisin expression. A. BAT expression of the rate-limiting enzyme in fatty acid oxidation, *Cpt1α* and BAT weight at sacrifice (g). B. Skeletal muscle *Fndc5* gene expression and plasma irisin. Data are presented as mean ± SEM, n = 8-12/group. One-way ANOVA with Tukey test for multiple comparisons were performed. *P<0.05 from HFHS control.
(TIF)

S8 Fig Core body temperature and total body mass-adjusted calorimetry. A. Core body temperature was measured at ambient room temperature (22°C) after 8 weeks on indicated diets, prior to cold exposure. Shaded areas indicate the dark cycle. B-D. Indirect calorimetry was performed as described in the experimental procedures section during an ambient temperature (22°C) and moderate chronic cold (14°C) exposure after 6 and 9 weeks on indicated diets, respectively. B. Body mass-adjusted oxygen consumption (VȮ_2_). C. Body mass-adjusted carbon dioxide production (VCȮ_2_). D. Body mass-adjusted heat production (kcal/kg/hour). Data are presented as mean ± SEM, n = 4/group. Data were analyzed using a mixed linear model with a compound symmetry covariance structure. ^#^P<0.05 from 22°C, *P<0.05 from HFHS control, **P<0.05 from chow.
(TIF)

S1 Table HFHS-based diet composition. (DOCX)

## Abbreviations

*Adipoq*: adiponectin
*Adrb3*: adrenoreceptor beta 3
*Arg1*: arginase 1
BAT: brown adipose tissue
*Ccl2*: chemokine (C-C motif) ligand 2
*Cidea*: cell death-inducing DFFA-like effector A
CLA: conjugated linoleic acid
*Cpt1*: carnitine palmitoyl transferase 1
CR: caloric restriction
*Egr2*: early growth response 2
EWAT: epididymal white adipose tissue
FAO: fatty acid oxidation
*Fndc5*: fibronectin type III domain-containing protein 5
GTT: glucose tolerance test
HFHS: high fat high sucrose diet
ITT: insulin tolerance test
IWAT: inguinal white adipose tissue
LDLR: low-density lipoprotein receptor
MetS: metabolic syndrome
*Nos2*: inducible nitric oxide synthase
*Ppar*: peroxisome proliferator activated receptor
RQ: respiratory quotient
*Slc2a2*: solute carrier family 2 member 2
*Tnf*: tumor necrosis factor
*Ucp1*: uncoupling protein 1
WAT: white adipose tissue
