# Supplementary figures and images for: Metabolically distinct weight loss by 10,12 CLA and caloric restriction highlight the importance of subcutaneous white adipose tissue for glucose homeostasis in mice
Source: PLoS One. 2017 Feb 28;12(2):e0172912. doi: 10.1371/journal.pone.0172912 (PMC5330530; doi:10.1371/journal.pone.0172912)

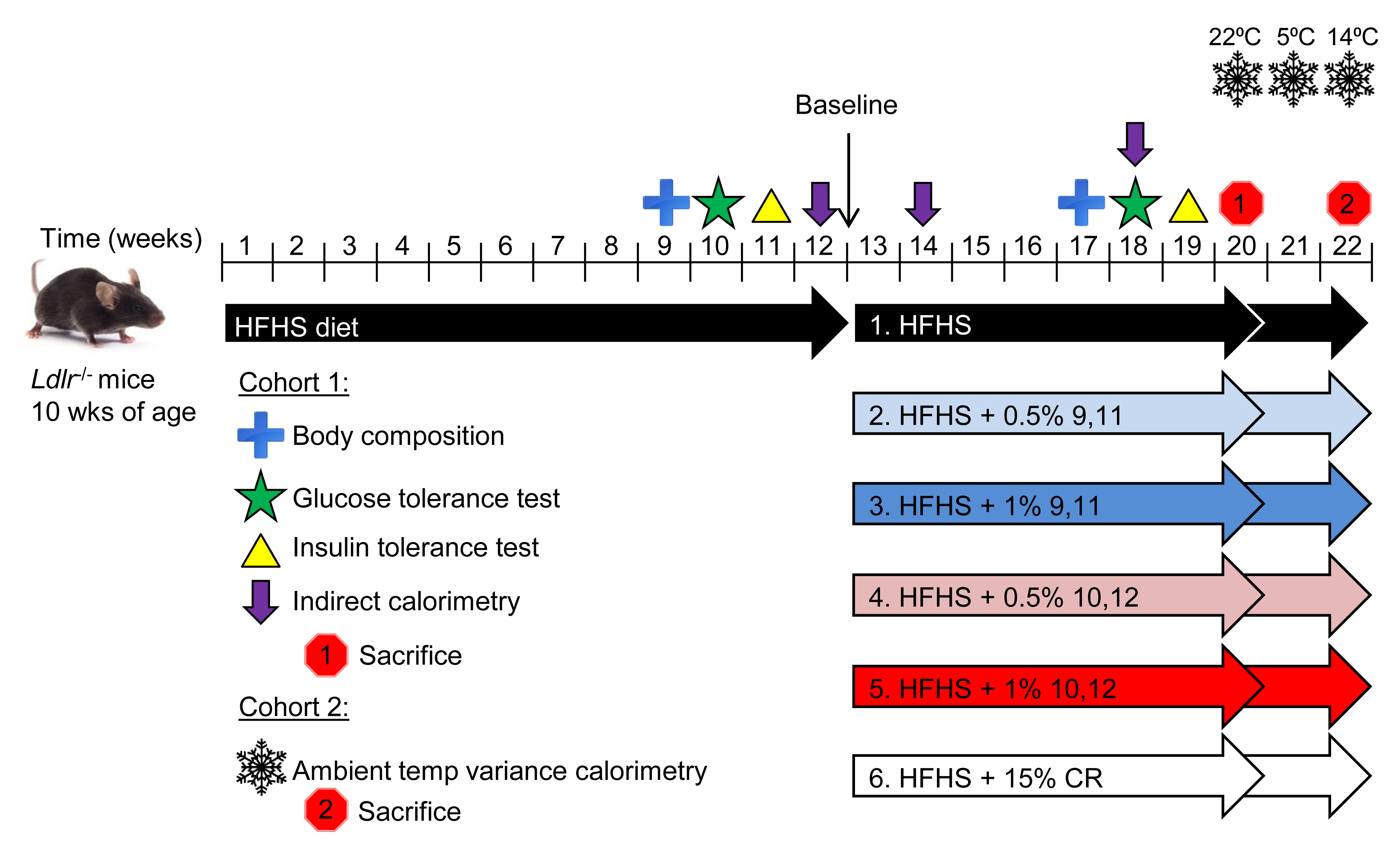

Supplement: S1 Fig — Two separate cohorts of male Ldlr-/- mice were fed a HFHS diet for 12 weeks, then continued on the HFHS diet with or without the addition of 0.5% 9,11 CLA, 1% 9,11 CLA, 0.5% 10,12 CLA, 1% 10,12 CLA, or 15% CR for an additional 8–10 weeks. Mice in cohort 1 underwent body composition, glucose tolerance testing, insulin tolerance testing, and indirect calorimetry as indicated on the timeline prior to sacrifice after 8 weeks on test diets (n = 10–15 mice/group). Mice in cohort 2 were implanted with a temperature transponder after 6 weeks on test diets. Following a 2-week recovery, indirect calorimetry was measured continuously during exposures to three separate ambient temperatures: (1) normal ambient room temperature (22°C for 3 days), (2) an acute cold challenge (5°C for 6 hours), and (3) a chronic cold challenge (14°C for 3 days) (n = 4 mice/group). A group of lean Ldlr-/- mice consuming normal rodent chow were used as a control group in cohort 2. (TIF) [file pone.0172912.s001.tif]

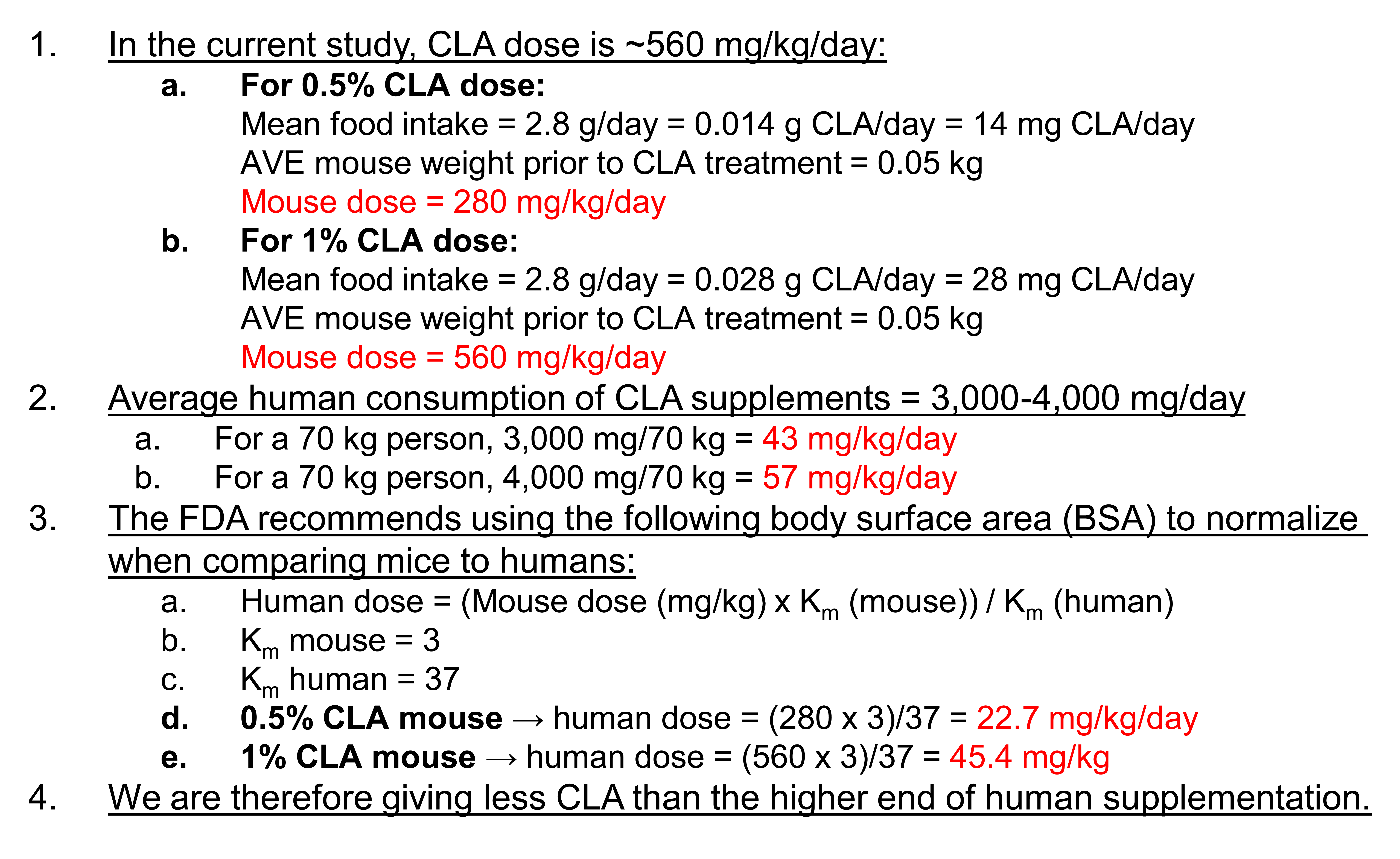

Supplement: S2 Fig — Representative calculations using a body surface area normalization method (Reagan-Shaw et al., [20]) to compare human and mouse CLA dose equivalencies. (TIF) [file pone.0172912.s002.tif]

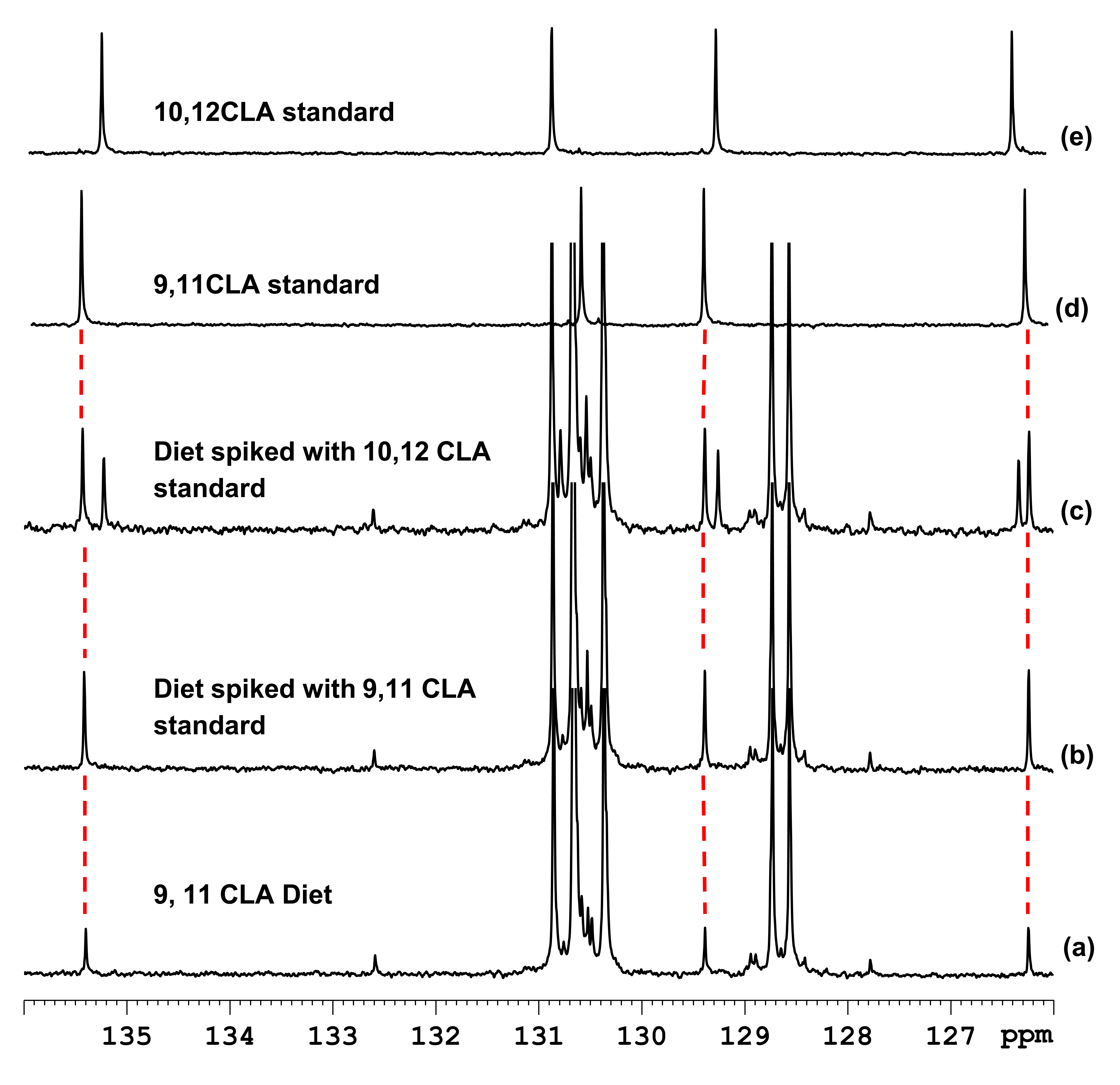

Supplement: S3 Fig — Parts of 13C NMR spectra of (a) 9,11 CLA diet; (b) 9,11 CLA diet spiked with 9,11 CLA standard; (c) 9,11 CLA diet spiked with 10,12 CLA standard; (d) 9,11 CLA standard; and (e) 10, 12 CLA standard. 13C chemical shifts of the four olefinic carbons were used to identify the specific CLA, unambiguously. The spectra were obtained on a Bruker Avance III 800 MHz spectrometer. (TIF) [file pone.0172912.s003.tif]

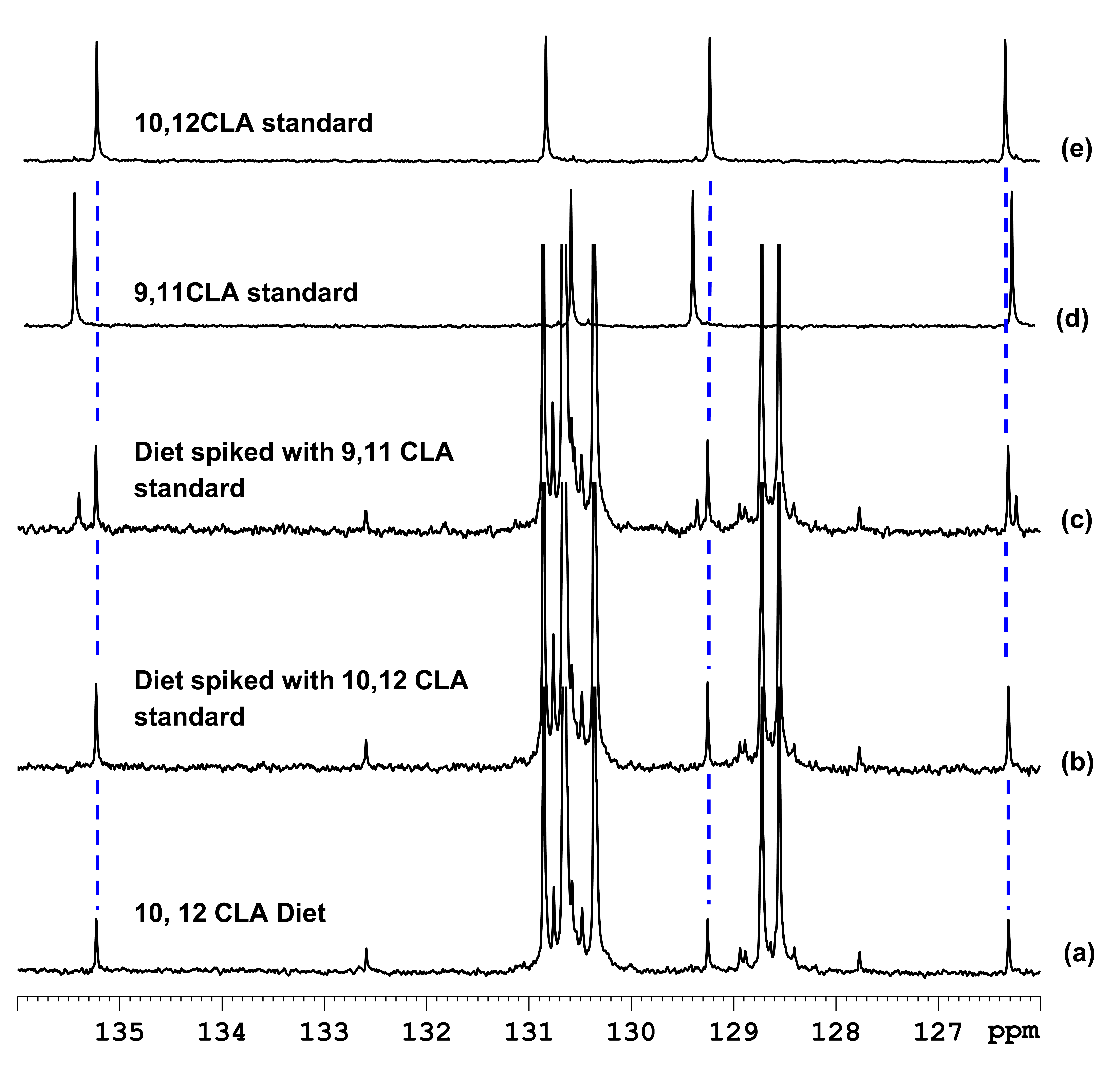

Supplement: S4 Fig — Parts of 13C NMR spectra of (a) 10,12 CLA diet; (b) 10,12 CLA diet spiked with 10,12 CLA standard; (c) 10,12 CLA diet spiked with 9,11 CLA standard; (d) 9,11 CLA standard; and (e) 10, 12 CLA standard. 13C chemical shifts of the four olefinic carbons were used to identify the specific CLA, unambiguously. The spectra were obtained on a Bruker Avance III 800 MHz spectrometer. (TIF) [file pone.0172912.s004.tif]

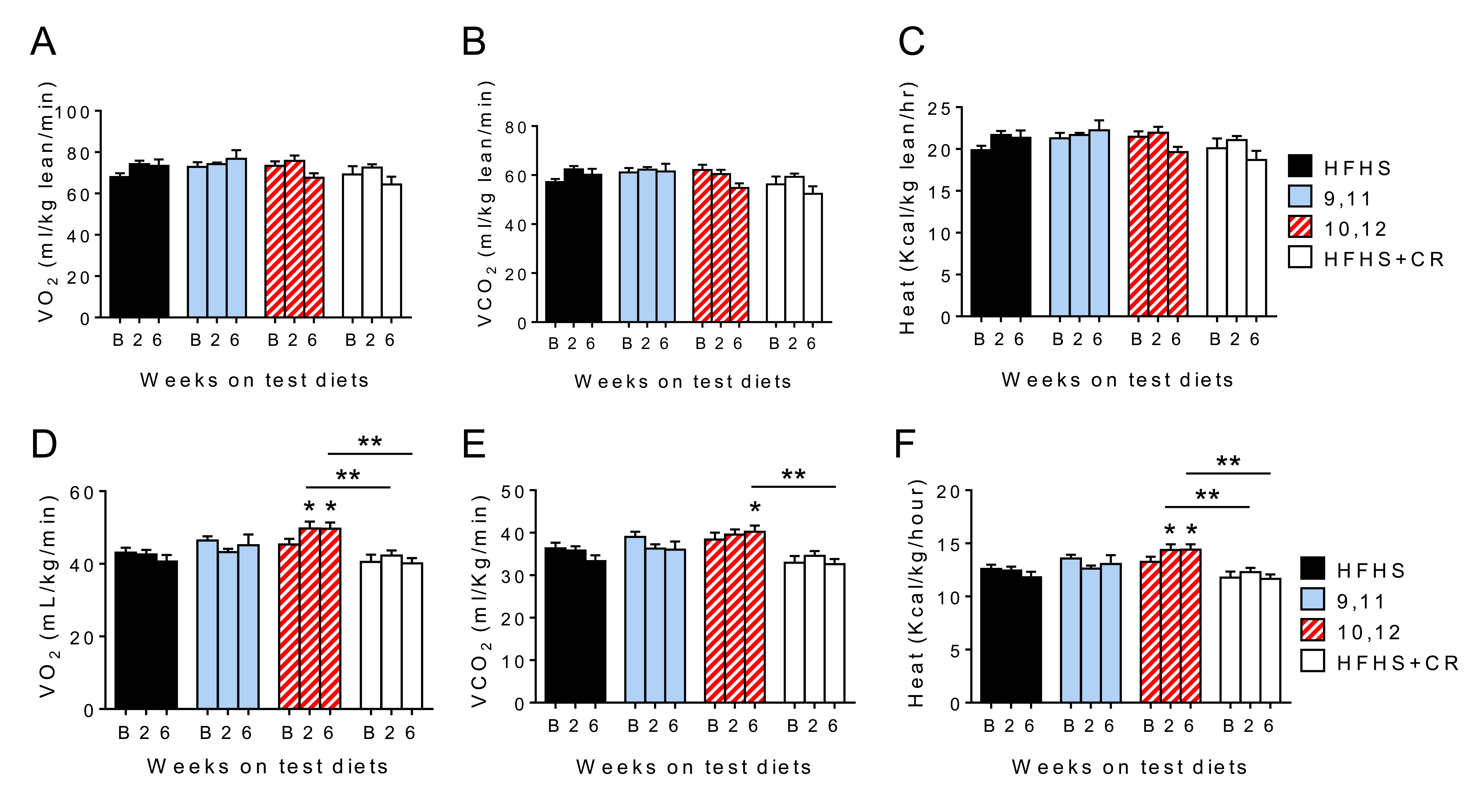

Supplement: S5 Fig — Indirect calorimetry was performed as described in the experimental procedures section at baseline (B), 2 weeks, and 6 weeks after the introduction of the test diets. A. Lean body mass-adjusted oxygen consumption (VȮ2), B. carbon dioxide production (VCȮ2), and C. heat production (kcal/kg/hour). D. Total body mass-adjusted oxygen consumption, E. carbon dioxide production, and F heat production (kcal/kg lean/hour). Data are presented as mean ± SEM, n = 8-12/group. Data were analyzed using a mixed linear model with a compound symmetry covariance structure. *P<0.05 from HFHS control, **P<0.05 from CR. (TIF) [file pone.0172912.s005.tif]

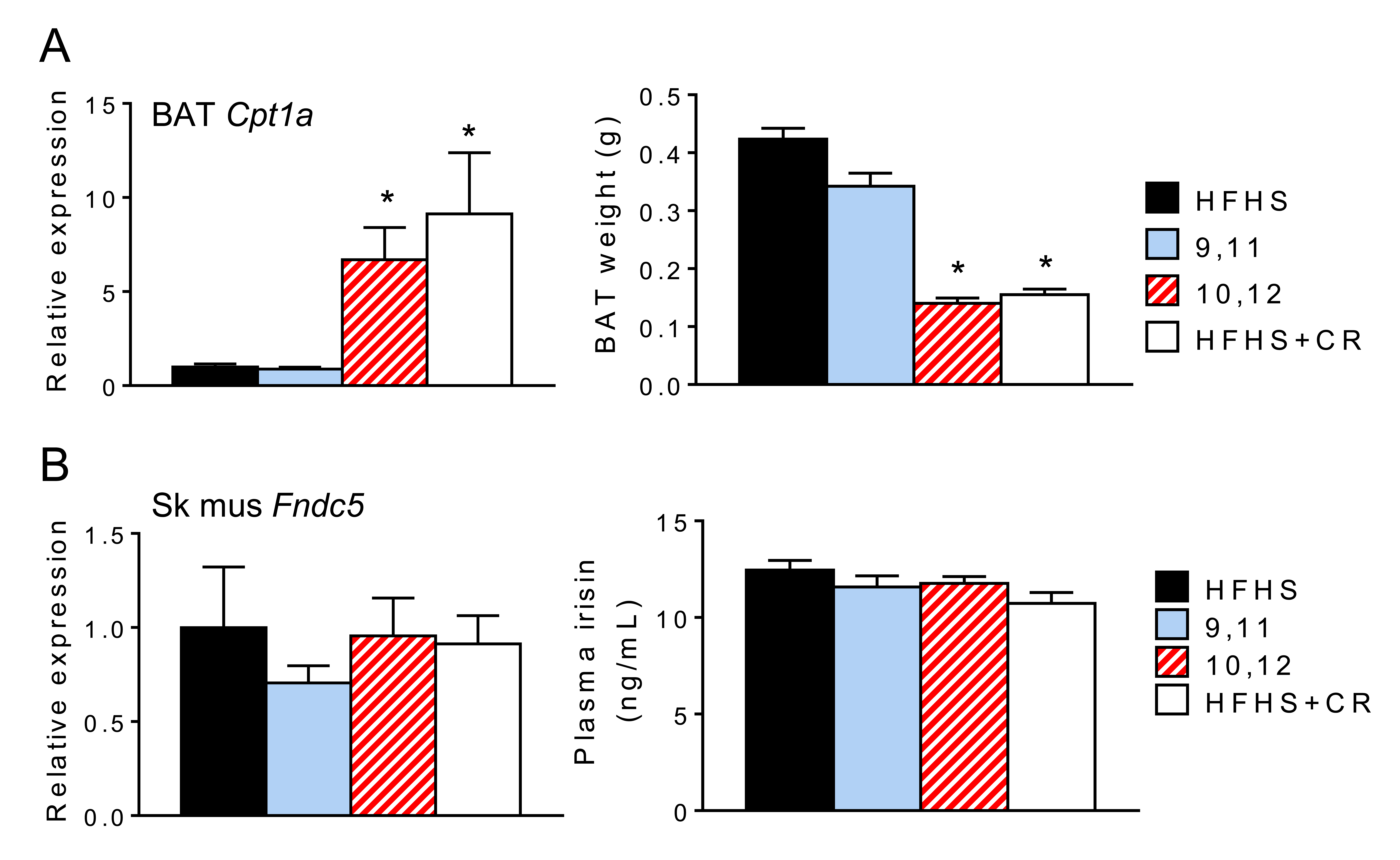

Supplement: S7 Fig — A. BAT expression of the rate-limiting enzyme in fatty acid oxidation, Cpt1α and BAT weight at sacrifice (g). B. Skeletal muscle Fndc5 gene expression and plasma irisin. Data are presented as mean ± SEM, n = 8-12/group. One-way ANOVA with Tukey test for multiple comparisons were performed. *P<0.05 from HFHS control. (TIF) [file pone.0172912.s007.tif]

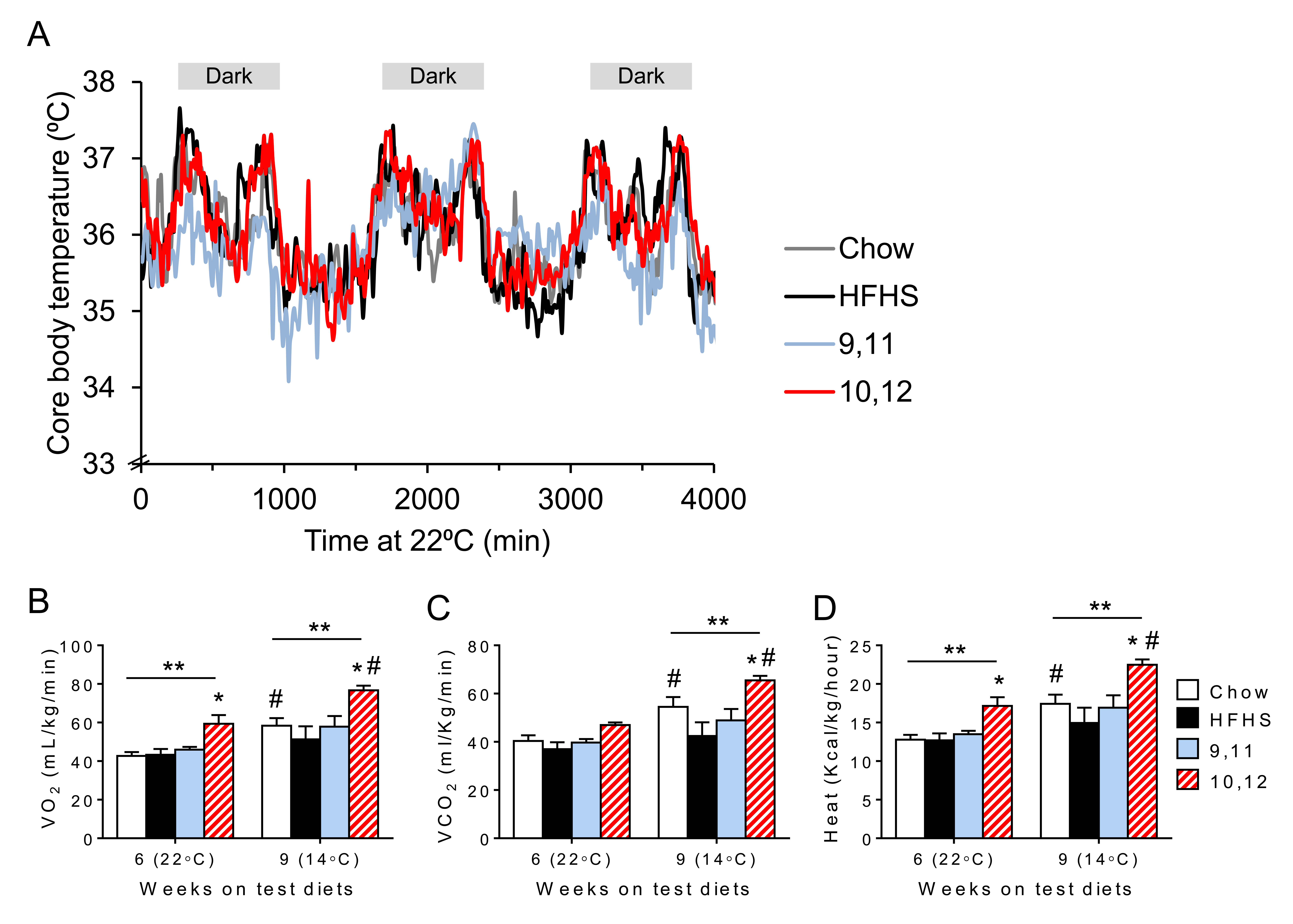

Supplement: S8 Fig — A. Core body temperature was measured at ambient room temperature (22°C) after 8 weeks on indicated diets, prior to cold exposure. Shaded areas indicate the dark cycle. B-D. Indirect calorimetry was performed as described in the experimental procedures section during an ambient temperature (22°C) and moderate chronic cold (14°C) exposure after 6 and 9 weeks on indicated diets, respectively. B. Body mass-adjusted oxygen consumption (VȮ2). C. Body mass-adjusted carbon dioxide production (VCȮ2). D. Body mass-adjusted heat production (kcal/kg/hour). Data are presented as mean ± SEM, n = 4/group. Data were analyzed using a mixed linear model with a compound symmetry covariance structure. #P<0.05 from 22°C, *P<0.05 from HFHS control, **P<0.05 from chow. (TIF) [file pone.0172912.s008.tif]
