# Supplementary material for: Metabolically distinct weight loss by 10,12 CLA and caloric restriction highlight the importance of subcutaneous white adipose tissue for glucose homeostasis in mice
Source: PLoS One. 2017 Feb 28;12(2):e0172912. doi: 10.1371/journal.pone.0172912 (PMC5330530; doi:10.1371/journal.pone.0172912)
Supplement: S1 Table — (DOCX) [file pone.0172912.s009.docx]

**S1 Table. HFHS-based diet composition**

|  | **Test diets** | | |
| --- | --- | --- | --- |
|  | **HFHS** | **HFHS +**  **1% 9,11 CLA** | **HFHS +**  **1% 10,12 CLA** |
| **Macronutrients (g/kg diet)** | | | |
| Protein | 205 | 205 | 205 |
| Fat | 360 | 360 | 360 |
| Fiber | 0 | 0 | 0 |
| Ash | 35 | 35 | 35 |
| Carbohydrate | 362 | 361 | 361 |
| Monosaccharides | 0.7 | 0.9 | 0.9 |
| Disaccharides | 243 | 243 | 243 |
| Polysaccharides | 113 | 111 | 111 |
| **Amino acids (g/kg diet)** |  |  |  |
| Alanine | 5.3 | 5.3 | 5.3 |
| Arginine | 7.3 | 7.3 | 7.3 |
| Aspartic acid | 12.8 | 12.8 | 12.8 |
| Cysteine | 0.6 | 0.6 | 0.6 |
| Glutamic acid | 40.5 | 40.6 | 40.6 |
| Glycine | 4.9 | 4.9 | 4.9 |
| Histidine | 5.5 | 5.5 | 5.5 |
| Isoleucine | 10.9 | 11.0 | 11.0 |
| Leucine | 16.6 | 16.6 | 16.6 |
| Lysine | 14.8 | 14.8 | 14.8 |
| Methionine | 7.1 | 7.1 | 7.1 |
| Phenylalanine | 8.9 | 8.9 | 8.9 |
| Proline | 20.5 | 20.5 | 20.5 |
| Serine | 11.4 | 11.4 | 11.4 |
| Threonine | 8.7 | 8.7 | 8.7 |
| Tryptophan | 2.2 | 2.2 | 2.2 |
| Tyrosine | 11.4 | 11.4 | 11.4 |
| Valine | 13.0 | 13.0 | 13.0 |
| **Fatty acids (g/kg diet)** | | | |
| SFA | 140 | 136.6 | 136.6 |
| Monounsaturated | 161 | 157 | 157 |
| Polyunsaturated | 40.1 | 39 | 39 |
| C18:2 Linoleic acid | 36.5 | 35.5 | 35.5 |
| c9,t11 CLA | - | 10 | - |
| t10,c12 CLA | - | - | 10 |
| C18:3 Linolenic acid | 3.6 | 3.5 | 3.5 |
| **Total fatty acids:** | **381.2** | **381.6** | **381.6** |
| **Minerals (g/kg diet)** | | | |
| Calcium | 5.6 | 5.6 | 5.6 |
| Chloride | 0.86 | 0.86 | 0.86 |
| Copper | 0.0036 | 0.0036 | 0.0036 |
| Chromium | 0.00041 | 0.00041 | 0.00041 |
| Fluoride | 0.011 | 0.011 | 0.011 |
| Iodine | 0.00031 | 0.00031 | 0.00031 |
| Iron | 0.0407 | 0.0408 | 0.0408 |
| Magnesium | 0.49 | 0.49 | 0.49 |
| Manganese | 0.0466 | 0.0467 | 0.0467 |
| Phosphorus | 5.8 | 5.8 | 5.8 |
| Potassium | 5.6 | 5.6 | 5.6 |
| Selenium | 0.00021 | 0.00022 | 0.00022 |
| Sodium | 0.570 | 0.571 | 0.571 |
| Sulfur | 0.667 | 0.668 | 0.668 |
| Zinc | .0215 | 0.0216 | 0.0216 |
| **Vitamins (g/kg unless noted otherwise)** | | | |
| Choline | 1.147 | 1.143 | 1.143 |
| Folic acid | 0.00075 | 0.00075 | 0.00075 |
| Niacin | 0.015 | 0.015 | 0.015 |
| Pantothenic acid | 0.0055 | 0.0055 | 0.0055 |
| Pyridoxine | 0.0041 | 0.0041 | 0.0041 |
| Riboflavin | 0.0022 | 0.0023 | 0.0023 |
| Thiamin | 0.003 | 0.003 | 0.003 |
| Vitamin A (IU/kg) | 3158 | 3162 | 3162 |
| Vitamin B_12_ (mcg/kg) | 40 | 40 | 40 |
| Vitamin D_2_ (IU/kg) | 999 | 1000 | 1000 |
| Vitamin E (IU/kg) | 25.7 | 25.6 | 25.6 |
| Vitamin K_3_ (Menadione) | 0.00052 | 0.00052 | 0.00052 |
